# Native and Non-Native Supergeneralist Bee Species Have Different Effects on Plant-Bee Networks

**DOI:** 10.1371/journal.pone.0137198

**Published:** 2015-09-10

**Authors:** Tereza C. Giannini, Lucas A. Garibaldi, Andre L. Acosta, Juliana S. Silva, Kate P. Maia, Antonio M. Saraiva, Paulo R. Guimarães, Astrid M. P. Kleinert

**Affiliations:** 1 Instituto de Biociências, Universidade de São Paulo, São Paulo, São Paulo, Brazil; 2 Instituto Tecnológico Vale Desenvolvimento Sustentado, Belém, Pará, Brazil; 3 Sede Andina, Universidad Nacional de Río Negro (UNRN) and Consejo Nacional de Investigaciones Científicas y Técnicas (CONICET), San Carlos de Bariloche, Río Negro, Argentina; 4 Instituto Federal de Educação, Ciência e Tecnologia de Mato Grosso, Cuiabá, Mato Grosso, Brazil; 5 Escola Politécnica, Universidade de São Paulo, São Paulo, São Paulo, Brazil; University of Cologne, GERMANY

## Abstract

Supergeneralists, defined as species that interact with multiple groups of species in ecological networks, can act as important connectors of otherwise disconnected species subsets. In Brazil, there are two supergeneralist bees: the honeybee *Apis mellifera*, a non-native species, and *Trigona spinipes*, a native stingless bee. We compared the role of both species and the effect of geographic and local factors on networks by addressing three questions: 1) Do both species have similar abundance and interaction patterns (degree and strength) in plant-bee networks? 2) Are both species equally influential to the network structure (nestedness, connectance, and plant and bee niche overlap)? 3) How are these species affected by geographic (altitude, temperature, precipitation) and local (natural vs. disturbed habitat) factors? We analyzed 21 plant-bee weighted interaction networks, encompassing most of the main biomes in Brazil. We found no significant difference between both species in abundance, in the number of plant species with which each bee species interacts (degree), and in the sum of their dependencies (strength). Structural equation models revealed the effect of *A*. *mellifera* and *T*. *spinipes*, respectively, on the interaction network pattern (nestedness) and in the similarity in bee’s interactive partners (bee niche overlap). It is most likely that the recent invasion of *A*. *mellifera* resulted in its rapid settlement inside the core of species that retain the largest number of interactions, resulting in a strong influence on nestedness. However, the long-term interaction between native *T*. *spinipes* and other bees most likely has a more direct effect on their interactive behavior. Moreover, temperature negatively affected *A*. *mellifera* bees, whereas disturbed habitats positively affected *T*. *spinipes*. Conversely, precipitation showed no effect. Being positively (*T*. *spinipes*) or indifferently (*A*. *mellifera*) affected by disturbed habitats makes these species prone to pollinate plant species in these areas, which are potentially poor in pollinators.

## Introduction

Supergeneralist species, defined as species that interact with multiple groups of species, are considered key species in interaction networks because they act as important connectors of species subsets that otherwise would be unconnected [1,2,3]. The selective removal of species with large number of interactions enhances the fragility of networks [4] and, for the specific case of pollinators, it may affect plant diversity [5]. Thus, it is important to understand the role of these species in interaction networks, especially considering the rapidly changing conditions of their habitats.

Global changes, mainly the presence of invasive species, climate changes, and habitat disturbance, exert important influences on interaction networks [6,7]. First, invasive species, even when acting as supergeneralists, can exhibit a different role in interaction networks, presenting a disruptive effect, modifying the strength of interactions, and decreasing the connectivity among native species, with detrimental consequences to their interacting partners [8,9,10] (but see exceptions regarding alien plants in [11,12,13]). Theoretical studies exploring the drivers behind network invasion showed that large [14] and more generalist [14,15] species are more successful invaders, whereas webs relatively easy to invade have low connectance and greater number of species [15]. Second, climate change, with increasing variability in temperature and precipitation, appears to have a more moderate effect on invasive species [16,17] and, in some cases, leads to a homogenization of interaction networks due to the expansion of generalists [18]. It can also disrupt interactions themselves since partner species may disperse differently when seeking for new habitats [19,20,21]. Third, disturbed habitats can be better tolerated by generalist species than specialized ones [22,23] and are more likely to facilitate the settlement of invasive species [24,25,26,27,28], also changing the network structure due to species loss and reorganization of interaction patterns [29]. Thus, complex abiotic-biotic features appear to drive species interactions.

**Fig 1:**
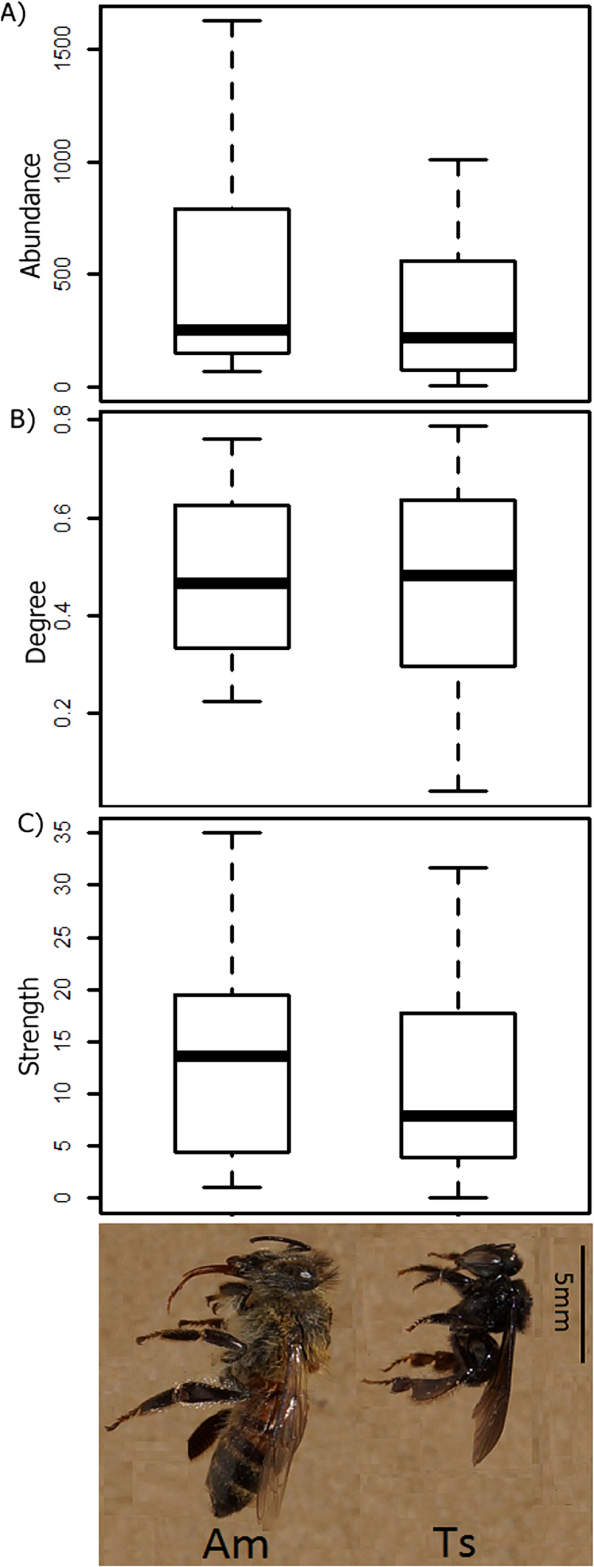
A) Abundance, B) Degree (number of interactions), and C) Strength of *Apis mellifera* (Am) and *Trigona spinipes* (Ts) in Brazilian weighted plant-bee networks. There is no significant difference between the variables (t-test; *P*> 0.05). The horizontal line within each box is the median, and the lower and upper limits of the box define the 25th and 75th percentiles, respectively. The lower and upper whiskers define the 10th and 90th percentiles, respectively. Photo by Adrian Gonzalez and Sheina Koffler.

Plant-bee interaction networks represent an important case study because pollination is a key ecosystem service [30,31] and multiple drivers related to rapid global change are affecting pollinators worldwide [32,33]. In Brazil, there are two well-known supergeneralist bee species playing a crucial role in interaction networks [34,35,36,37,38]. One is an invasive bee species, *Apis mellifera* Linnaeus 1758 (Apidae), introduced in the 1950s and currently scattered in all Brazilian regions [39]; the other is *Trigona spinipes* Fabricius 1793 (Apidae), a smaller native social stingless bee (Fig 1). Both were recently quoted as pollinators of some crop species [40] but their role as actual pollinator of native flora and competitor for resources has to be clarified [41,42,43].

The role of supergeneralist species has being debated, and they are suggested to be key elements of mutualistic networks ([44] but see [45]), potentially shaping evolutionary dynamics [3] and being vulnerable to human impact [46]. However, the simultaneous presence of one native and one invasive bee species in the same interaction networks arises intriguing questions and, to our knowledge, this is the first attempt to analyze a situation like this.

In this work, our aim is to analyze the role of invasive *A*. *mellifera* and native *T*. *spinipes* in Brazilian plant-bee interaction networks by addressing three main questions: 1) Do both supergeneralist species have similar interaction patterns in pollination networks? 2) Are both species equally influential to the network structure? 3) How are these species affected by geographic (climate) and local (natural vs. disturbed habitats) factors? Answering these questions would represent an additional step in understanding the effect of these supergeneralist bees on interaction networks.

## Materials and Methods

We selected surveys of interactions between bees and plants conducted in Brazil, which comprised at least one year of monthly observations. All surveys followed the procedure suggested by [47], in which the researcher spends a fixed amount of time (3 to 5 minutes) on each flowering plant or patch along an established transect and collects all observed bees using an entomological net (see S1 Table, for data sources). All surveys were performed more than 3.7 km apart (except one) and we assumed that sample sites were independent from each other. Since data on species interactions prior to the invasion of *Apis mellifera* is not available, the analysis presented here is only regarding the post-establishment communities. *A*. *mellifera* and *T*. *spinipes* were both collected in all surveys analyzed. In most surveys, the type of interactions among the bees and flowers were not detailed and could include effective pollination and (or) foraging for nectar, pollen, or oil. We updated the bee taxonomic names according to Moure’s Bee Catalogue website (http://moure.cria.org.br/) and plants according to W3Tropic of Missouri Botanical Garden (http://www.tropicos.org/).

We built the interaction network of each survey, which is a set of nodes (species) connected through links, with each link representing an observed interaction [48]. We used the surveys that presented the number of bees sampled per plant to build weighted networks. In this case, a positive integer indicated an interaction, representing the number of times a pair of species interacts [49], that means, the frequency of interaction instead of presence/absence interaction. To reduce the differences in overall sample sizes between different networks we normalized the data, since this procedure better reflects the role of each species in the network than the raw data, avoiding any bias in the process analysis [50]. To compute the species-level metrics we standardized the matrix in such way that if a(i,j) represents the number of interactions of the bee i with plant j, then the w(i,j) will be equal to a(i,j) divided by the sum of all interactions of species i if we are computing the species-level metrics for bees, and w(i,j) will be equal to a(i,j) divided by the sum of interactions of species j if we are computing the species-level metrics for plants [51].

To answer the first question (do both species have similar abundance and interaction patterns in plant-bee networks?), we performed a paired t-test using three features: abundance, degree, and strength of *A*. *mellifera* and *T*. *spinipes*. The abundance of each bee species is the sum of all individual bees of that species that were captured in every plant during each survey. Degree is the number of links per species [44], i.e., the number of plant species with which each bee species interacts; thus, normalized degree is the proportion of species that a certain species interacts with, out of the total possible interaction in the network [52]. Strength is measured as the relative frequency of visits and represents the sum of dependencies of a species [51], which express the proportion of all observed interactions for each species with other species on network. Dependence is calculated as d_ij_ = N_ij_/N_i_, where N_ij_ represents the number of interactions observed between the species i and j, and N_i_ the total number of interactions identified to the plant species i [53].

To answer the second question (are both species equally influential to the network structure?), we used three metrics at the network level: nestedness, connectance, and bee and plant niche overlap. The nestedness metric used was NODF (Nestedness based on Overlap and Decreasing Fill, following [54]), which describes a pattern of interaction in which specialists interact with species that form perfect subsets of the species with which generalists interact [48]. A nested structure implies the existence of a group of highly connected species [55], which minimizes competition and increases the number of coexisting species ([56], but see [57]). Nestedness also makes the community more robust to extinction [5, 58] and habitat loss [59]. Connectance is the realized proportion of possible interactions [48,60]. Niche overlap occurs when two organismic units use the same resources (or other environmental variable) and indicates the resemblance on resource utilization, for example, the likeliness of plant species set used by different bee species. In other words, it estimates the mean similarity in interaction pattern between species of the same level [60], and is calculated using Horn’s index [61]. Values near ‘zero’ indicate no common use of partners and values equal ‘one’ indicate perfect niche overlap [60].

To answer the third question (are both supergeneralist species affected by geographic factors?), we characterized the environmental features using the geographic coordinates of each survey and three environmental layers from the bioclimatic dataset of [62]: altitude, annual mean temperature, and annual precipitation. We extracted the information about these features for each locality surveyed using ArcGIS 10 (Esri Inc.). As some of the surveys were performed in the 1980 and 1990 decades, we considered the information about habitat disturbance (natural vs. modified) provided by the author of the study. We considered modified habitats those surveys conducted on agro-ecosystems or in urban areas.

We used the ‘specieslevel’ function available in the ‘bipartite’ package [60] to calculate the metrics that describe the role of each supergeneralist species in the interaction networks (degree and strength) and the ‘networklevel’ function, also available in ‘bipartite’ package [60], to calculate the metrics that describe the network structure (nestedness, connectance, and niche overlap), both within R [63]. The ‘specieslevel’ function required the ‘sna’ package [64]. As there is a high correlation between various network metrics [60], we measured the correlation between all of the variables. We also added the values of bee and plant richness and bee abundance of each network to evaluate their correlation with the other estimates.

We estimated one linear structural equation model (‘sem’ package) [65] using R [63] to test a conceptual model for plant-bee interaction networks in Brazil. Structural equation models (SEM) are well suited to estimate conceptual frameworks when there are several predictor variables and direct and indirect connections, such as in our case [66,67]. Specifically, the model tested the effect of the degree and strength of both supergeneralist bees on nestedness and on plant and bee niche overlap (question 2). In addition, the model tested the effect of environmental variables (annual mean temperature and annual precipitation) and disturbance (natural vs. modified habitats) on the degree and strength of *A*. *mellifera* and *T*. *spinipes* (question 3). In this sense, strength and degree of *A*. *mellifera* and *T*. *spinipes* acted both as predictor (question 2) and response (question 3) variables (see [68] for details on coefficient estimations). Because of the high Pearson's correlation coefficient among the strength of *A*. *mellifera* and the strength of *T*. *spinipes* (Pearson's *r* is 0.66; see S2 Table), we included their co-variation in the structural equation model. Moreover, in the subsequent analyses, we discarded connectance due to its high correlation with nestedness (Pearson's *r* is 0.97; see S2 Table) and discarded altitude due to its high correlation with temperature (Pearson's *r* is -0.93; see S2 Table). In all cases, we presented standardized coefficients to allow comparison of the intensity of different relationships. All variables met the assumptions of linearity and normality (Kolmogorov-Smirnov test; Type I error rate = 0.05).

## Results

We found 21 surveys presenting the number of each bee species found on a plant species (weighted interaction networks) (see S1 Table for further details about data sources). From the 21 interaction networks, we obtained 980 species of bees and 1,246 of plants. There was a strong and positive correlation between the bee and plant richness across all sites (Pearson's *r* of 0.72, S2 Table).

The abundance, degree, and strength of both supergeneralist bees (*A*. *mellifera* and *T*. *spinipes*) showed no significant difference (t-test *P*>0.05) (Fig 1) (question 1). The interaction networks showed 48,212 bee individuals sampled, with 11,022 individuals of *A*. *mellifera* (23% of the total) and 12,938 of *T*. *spinipes* (27% of the total). The *A*. *mellifera* and *T*. *spinipes* degrees presented averages of 0.48 and 0.43 and strengths of 14 and 11, respectively (Fig 1). The strengths of *A*. *mellifera* and *T*. *spinipes* increased at sites with higher plant richness but were not associated with bee richness (Pearson's *r* of 0.79 and 0.65, respectively, S2 Table). In contrast, there was no correlation between the degree of *A*. *mellifera* and *T*. *spinipes*, and plant or bee richness (S2 Table). There was a positive correlation between the abundance and strength of *A*. *mellifera* and between the degree and strength of *T*. *spinipes*. Moreover, there was a positive correlation between the *T*. *spinipes* abundance and *T*. *spinipes* strength and degree (Pearson's *r* between 0.57 and 0.82, S2 Table). Interestingly, the *A*. *mellifera* abundance did not co-vary with the *T*. *spinipes* abundance (Pearson's *r* = 0.39), but both were positively correlated with the abundance of all other bees aggregated (Pearson's *r* of 0.59 and 0.62 for *A*. *mellifera* and *T*. *spinipes*, respectively, S2 Table).

**Fig 2:**
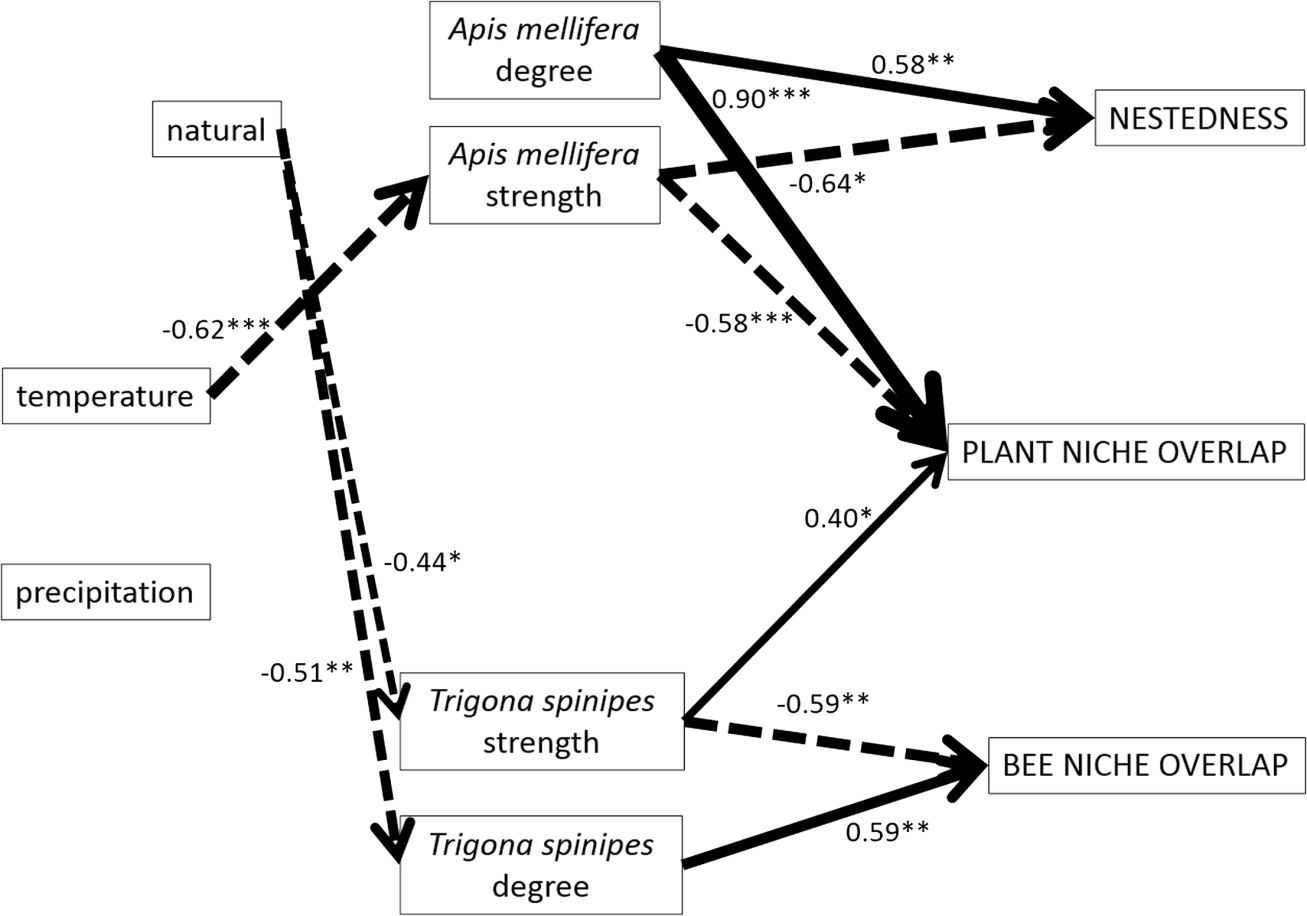
Conceptual model of the regulation of geographical variation in Brazilian weighted plant-bee networks tested by a structural equation model. Only significant effects are shown: *P<0.05; **P<0.01; ***P<0.001. The arrow size is proportional to the effect size (the intensity of relationship). The dotted arrows indicate negative effects. “Natural” refers to an undisturbed area (see Materials and Methods).

The structural equation model showed significant relationships among the proposed variables (question 2; Fig 2; RMSEA index = 0.34, Bentler CFI = 0.57). The among-site variation for the *A*. *mellifera* strength and degree had a strong and contrary impact on the network metrics (nestedness and plant niche overlap), with the first positive and the second negative (Fig 2). In contrast, *T*. *spinipes* presented lower effects, being related only to the plant and bee niche overlap (Fig 2). In particular, we found that the *A*. *mellifera* strength (negative) and degree (positive), and *T*. *spinipes* strength (positive) were associated with the plant niche overlap, whereas the *T*. *spinipes* strength (negative) and degree (positive) were associated with the bee niche overlap (Fig 2) (see also S1 Fig).

**Fig 3:**
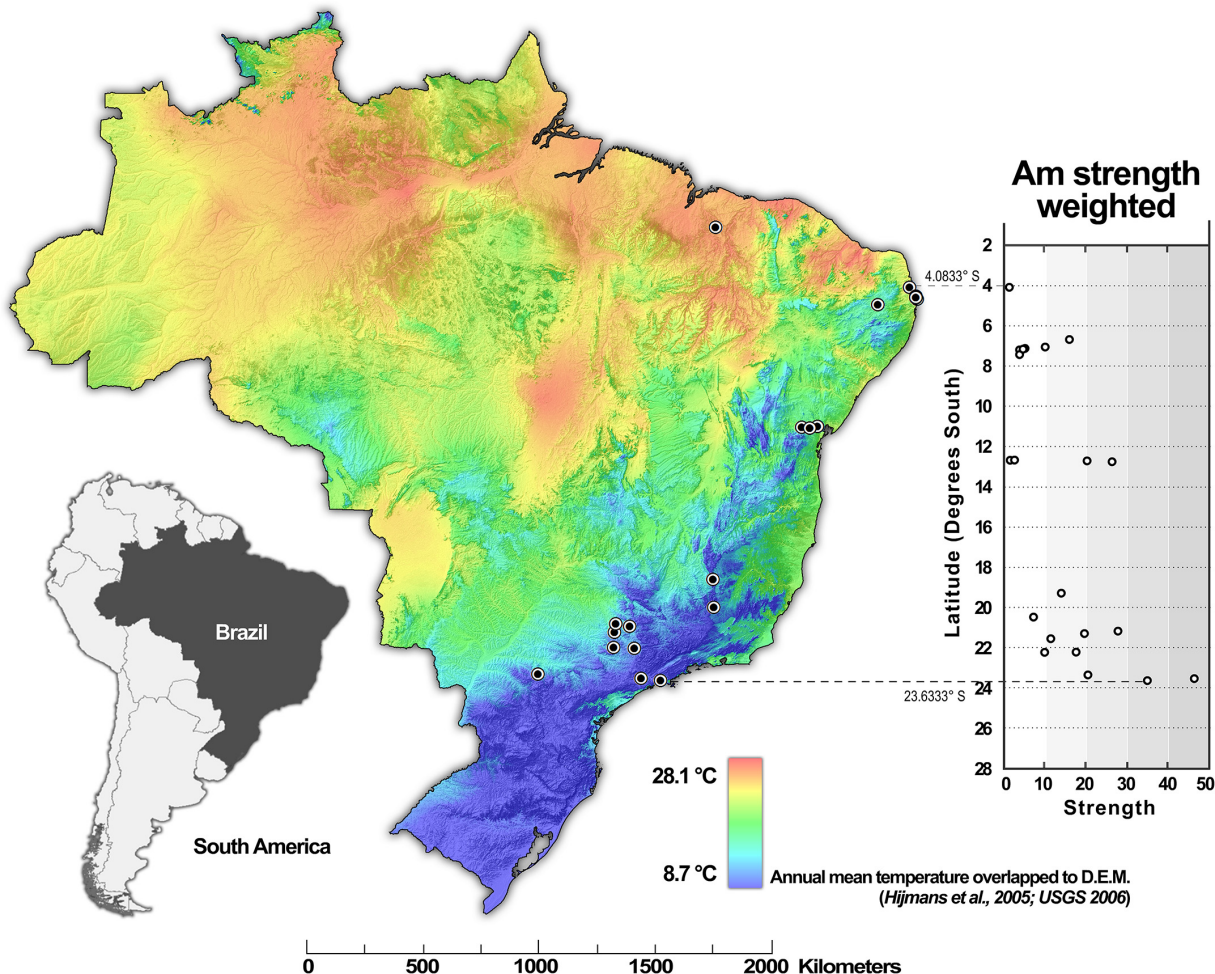
The strength of *Apis mellifera* (Am) decreases with temperature at lower latitudes in Brazilian weighted plant-bee networks. The strength of *Trigona spinipes* (Ts) was not included because it presented no significant relationship to temperature (see Fig 2) (DEM = Digital Elevation Model). Map was built using ArcGIS 10 (Esri Inc.).

Annual mean temperature was negatively associated with the *A*. *mellifera* strength only (question 3; Figs 2 and 3). Annual precipitation showed no effect on the metrics of either species (Fig 2). The local land-use influenced *T*. *spinipes* but did not influence *A*. *mellifera*. Specifically, the strength and degree of *T*. *spinipes* decreased in natural habitats in comparison to modified habitats (Fig 2).

## Discussion

Supergeneralist species, which interact with multiple groups of species and act as connectors of otherwise unconnected species, are important for maintaining the robustness of networks. In this study, we show that native and non-native supergeneralist bees, despite their similarities (question 1), exert different effects on interaction networks (question 2) and are affected differently by climate and landscape features (question 3).

The significant similarity of both supergeneralist bees, described here as their abundance, degree, and strength, is most likely due to their ability to occupy broad distributional ranges and the relative independence of cavities in which to build their nests. The higher number of interactions of some species may be, in many cases, associated with their abundances. There is a heated debate in the literature on the role abundance plays in structuring ecological networks [49,69,70]. In fact, models based on the neutral theory often predict the existence of highly connected species [71]. Nevertheless, under the assumption of neutrality, the most abundant species at a local scale is the result of ecological drift, which also predicts that most abundant species will vary across different sites. Therefore, it is not expected the same few species to be the dominant components of ecological assemblages in many different sites. In this sense, the dominance of these supergeneralist bees may be a consequence of their traits, which in turn, may also explain their ecological success. In spite of having different body sizes, both bee species present colonies with a very large number of individuals and were once considered "similar species" [72].

However, both species do not have the same effect on network structure since only *A*. *mellifera* showed a strong effect on nestedness, whereas *T*. *spinipes* was found to present a main effect on the bee niche overlap. The correlation between *A*. *mellifera* degree and nestedness was positive, suggesting that the higher the number of interactions of *A*. *mellifera*, the higher the nestedness. Nestedness describes a common topology where the most generalist species interact among them generating a core of interactions to which the rest of the species is attached, implying the existence of a relatively small group of highly connected species. In our case, *A*. *mellifera* seems to exert a positive effect on this type of topology. As already pointed, this supergeneralist species is fundamental to the maintenance of the whole network, since it participates on most of the links established with plant species. Also, the positive correlation with niche overlap suggests that the similarity of interaction patterns in each trophic level is directly related to the number of interactions showed by *A*. *mellifera* with its partners. Finally, it was already pointed by other authors that recent invasive species enter networks, interacting primarily with native generalists, which directly and rapidly increases nestedness [73]. Moreover, the dependence of plants on the new species may be lower than expected because other native pollinators most likely are more tightly associated with native plants. This could explain the opposite results obtained when analyzing the role of the degree (number of interactions) and strength (dependence of plants) of *A*. *mellifera* on nestedness. However, the long-term interactions between *T*. *spinipes* and other native bees could have resulted in a higher effect on the adaptation of other bees. Although *A*. *mellifera* appears to displace the other bees from plant resources, making them change their phenology [42], *T*. *spinipes* presents a more direct effect on them, since they display an aggressive behavior when interacting with other bees during foraging [74].

Our data also suggest that high temperature reduces the strength of *A*. *mellifera* and increases network nestedness and plant niche overlap, temperature being highly correlated to altitude. This result indicates that the higher the temperature, the lesser is the dependence of *A*. *mellifera* and also, the higher is the aggregation of generalist species in a core (nestedness) and the similarity between partner species (niche overlap). It was already demonstrated that the mean annual temperature positively influences *A*. *mellifera* nest density only up to values equal to 25°C, whereas higher temperatures produce an inverse effect [75]. In addition, during seasons with extremely high temperatures, the abundance of *A*. *mellifera* decreases locally [76]. It is likely that local bees are more adapted to severe environmental conditions, playing central roles in these networks and apparently displacing *A*. *mellifera*. However, it is important to notice that temperature was negatively correlated to plant richness, what could also be mediating these results. Unlike temperature, precipitation showed no significant effect on either species. However, our dataset did not include networks on the Amazon biome, an area of constant high rainfall in Brazil; thus, the effect of precipitation on the role of these bees remains unclear. Finally, we found no correlation between disturbed habitats and *A*. *mellifera*, while this variable was correlated positively with *T*. *spinipes* strength. The lack of correlation found on *A*. *mellifera* interaction pattern suggests that this invasive species is neither favored nor hindered by habitat degradation whereas the positive correlation between habitat degradation and *T*. *spinipes* strength may suggest that this species responds well to disturbances. This emphasizes the potential role of both species as pollinators of local plants in degraded areas, which typically have smaller pollinator diversity [77]. Our results are a good example of the effect of different habitats inducing different interactions.

In short, our results suggest that temperature has an important effect on *A*. *mellifera* and disturbance, on *T*. *spinipes*. Both species are correlated differently to networks, being *A*. *mellifera* more influential on network topology (nestedness and plant niche overlap) and *T*. *spinipes* more influential on the interaction patterns of plants and other bees (plant and bee niche overlap). Overall, our results suggest that highly generalist invasive species alter the structure of interaction networks, and act differently from other equally generalist species, but which are not exotic, i.e., those that participate on networks for a long-time period. These species may present different answers to global changes, with consequences for their interaction networks and to the ecosystem services delivered by them. Understanding these relationships more accurately could contribute to the establishment of conservation programs that address management and public policy, aiming to enhance the protection of pollinators.

## Supporting Information

S1 Fig Variation of a) Nestedness and degree of *Apis mellifera* (Am); b) Plant niche overlap and degree of *Apis mellifera* (Am); b) Nestedness and strength of *Apis mellifera* (Am); d) Plant niche overlap and strength of *Apis mellifera* (Am); e) Plant niche overlap and strength of *Trigona spinipes* (Ts); f) Bee niche overlap and strength of *Trigona spinipes* (Ts); g) Bee niche overlap and degree of *Trigona spinipes* (Ts). (DOCX)

S1 Table Data sources. (DOCX)

S2 Table Pearson's correlation coefficients between total number of individual sampled, total richness, and the network indexes employed in our study. (DOCX)

## References

[1] Olesen JM, Eskildsen LI, Venkatasamy S. Invasion of pollination networks on oceanic islands, importance of invader complexes and endemic super generalists. Divers Distrib 2002; 8: 181–192.

[2] Olesen JM, Bascompte J, Dupont YL, Jordano P. The modularity of pollination networks. PNAS 2007; 104: 19891–19896. 18056808 10.1073/pnas.0706375104 PMC2148393

[3] Guimarães PR Jr, Jordano P, Thompson JN. Evolution and coevolution in mutualistic networks. Ecol Lett 2011; 14: 877–885. 10.1111/j.1461-0248.2011.01649.x 21749596

[4] Solé RV, Montoya JM. Complexity and fragility in ecological networks. Proc R Soc Lond B 2001; 268: 2039–2045. 10.1098/rspb.2001.1767 PMC1088846 11571051

[5] Memmott J, Waser NM, Price MV. Tolerance of pollination networks to species extinctions. Proc R Soc B 2004; 271: 2605–2611. 15615687 10.1098/rspb.2004.2909 PMC1691904

[6] Burkle LA, Alarcón R. The future of plant-pollinator diversity: understanding interaction networks across time, space, and global change. Am J Bot 2011; 98: 528–538. 10.3732/ajb.1000391 21613144

[7] Valiente-Banuet A, Aizen MA, Alcantara JM, Arroyo J, Cocucci A, Galetti M et al Beyond species loss: the extinction of ecological interactions in a changing world. Functional Ecology 2015, 29: 299–307.

[8] Simberloff D, von Holle B. Positive interactions of nonindigenous species, invasional meltdown? Biol Invasions 1999; 1: 21–32.

[9] Traveset A, Richardson DM. Biological invasions as disruptors of plant reproductive mutualisms. TREE 2006; 21: 208–16. 16701087 10.1016/j.tree.2006.01.006

[10] Aizen MA, Morales CL, Morales JM. Invasive mutualists erode native pollination webs. Plos Biology 2008; 6: e31 10.1371/journal.pbio.0060031 18271628 PMC2235906

[11] Lopezaraiza-Mikel ME, Hayes RB, Whalley MR, Memmott J. The impact of an alien plant on a native plant-pollinator network: an experimental approach. Ecol Lett 2007; 10: 539–550. 17542933 10.1111/j.1461-0248.2007.01055.x

[12] Bartomeus I, Vilà M, Santamaría L. Contrasting effects of invasive plants in plant–pollinator networks. Oecologia 2008; 155, 761–770. 10.1007/s00442-007-0946-1 18188603

[13] Vilà M, Bartomeus I, Dietzsch AC, Petanidou T, Steffan-Dewenter I, Stout JC et al Invasive plant integration into native plant-pollinator networks across Europe. Proc R Soc Lond B 2009; 276: 3887–3893. 10.1098/rspb.2009.1076 PMC2817287 19692403

[14] Lurgi M, Galiana N, López BC, Joppa LN, Montoya JM. Network complexity and species traits mediate the effects of biological invasions on dynamic food webs. Frontiers in Ecology and Evolution 2014; 2: 1–11.

[15] Romanuk TN, Zhou Y, Brose U, Berlow EL, Williams RJ, Martinez ND. Predicting invasion success in complex ecological networks. Phil Trans R Soc B 2009; 364: 1743–1754. 10.1098/rstb.2008.0286 19451125 PMC2685429

[16] Goulson D. Effects of introduced bees on native ecosystems. Annu Rev Ecol Evol Syst 2003; 34: 1–26.

[17] Schweiger O, Biesmeijer JC, Bommarco R, Hickler T, Hulme PE, Klotz S. et al Multiple stressors on biotic interactions: how climate change and alien species interact to affect pollination. Biol Rev 2010; 85: 777–795. 10.1111/j.1469-185X.2010.00125.x 20184567

[18] Davey CM, Devictor V, Jonzen N, Lindstrom A, Smith HG. Impact of climate change on communities: revealing species' contribution. J Anim Ecol 2013; 82: 551–561. 10.1111/1365-2656.12035 23398634

[19] Memmott J, Craze PG, Waser NM, Price MV. Global warming and the disruption of plant-pollinator interactions. Ecol Lett 2007; 10: 710–717. 17594426 10.1111/j.1461-0248.2007.01061.x

[20] Schweiger O, Settele J, Kudrna O, Klotz S, Kuhn I. Climate change can cause spatial mismatch of trophically interacting species. Ecology 2008; 89: 3472–3479. 19137952 10.1890/07-1748.1

[21] Lurgi M, Lopez BC, Montoya JM. Novel communities from climate change. Philos Trans R Soc Lond B Biol Sci 2012; 367: 2913–2922. 23007079 10.1098/rstb.2012.0238 PMC3479747

[22] Clavel J, Julliard R, Devictor V. Worldwide decline of specialist species, toward a global functional homogenization? Front Ecol Environ 2011; 9: 222–228.

[23] Aizen MA, Sabatino M, Tylianakis JM. Specialization and rarity predict nonrandom loss of interactions from mutualist networks. Science 2012; 335: 1486–1489. 10.1126/science.1215320 22442482

[24] Marvier M, Kareiva P, Neubert MG. Habitat destruction, fragmentation, and disturbance promote invasion by habitat generalists in a multispecies metapopulation. Risk Analysis 2004; 24: 869–77. 15357806 10.1111/j.0272-4332.2004.00485.x

[25] Levine LJ, Adler PB, Yelenik SG. A meta-analysis of biotic resistance to exotic plant invasions. Ecol Lett 2004; 7: 975–989.

[26] Chytry M, Maskell LC, Pino J, Pysek P, Vilà M, Font X et al Habitat invasions by alien plants, a quantitative comparison among Mediterranean, subcontinental and oceanic regions of Europe. J Appl Ecol 2008; 45: 448–458.

[27] Wilsey BJ, Daneshgar PP, Polley HW. Biodiversity, phenology and temporal niche differences between native-and novel exotic-dominated grasslands. Perspect Plant Ecol 2011; 13: 265–276.

[28] Catford JA, Vesk PA, White MD, Wintle BA. Hotspots of plant invasion predicted by propagule pressure and ecosystem characteristics. Divers Distrib 2011; 17: 1099–1110.

[29] Spiesman BJ, Inouye BD. Habitat loss alters the architecture of plant–pollinator interaction networks. Ecology 2013; 94: 2688–2696. 24597216 10.1890/13-0977.1

[30] Costanza R, d’Arge R, Groot RS, Farber S, Grasso M, Hannon B, et al The value of the world’s ecosystem services and natural capital. Nature 1997; 387: 253–260.

[31] Costanza R, Groot R, Sutton P, Ploeg S, Anderson SJ, Kubiszewski I et al Changes in the global value of ecosystem services. Global Environ Change 2014; 26: 152–158.

[32] Potts SG, Biesmeijer JC, Kremen C, Neumann P, Schweiger O, Kunin WE. Global pollinator declines: trends, impacts and drivers. TREE 2010; 25: 345–353. 10.1016/j.tree.2010.01.007 20188434

[33] Lever JJ, van Nes EH, Scheffer M, Bascompte J. The sudden collapse of pollinator communities. Ecol Lett 2014; 17: 350–359. 10.1111/ele.12236 24386999

[34] Biesmeijer JC, Slaa EJ, Castro MS, Viana BF, Kleinert AMP, Imperatriz-Fonseca VL. Connectance of Brazilian social bee, food plant network is influenced by habitat, but not by latitude, altitude or network size. Biota Neotropica 2005; 5: 85–93.

[35] Freitas BM, Imperatriz-Fonseca VL, Quezada-Euan JJG, Medina LM, Kleinert AMP, Galetto L et al Diversity, threats and conservation of native bees in the Neotropics. Apidologie 2009; 40: 332–346.

[36] Santos GMM, Aguiar CML, Genini J, Martins CF, Zanella FCV, Mello MAR. Invasive Africanized honeybees change the structure of native pollination networks in Brazil. Biol Invasions 2012; 14: 2369–2378.

[37] Kleinert AMP, Giannini TC. Generalist bee species on Brazilian bee-plant interaction networks. Psyche 2012; 291519: 1–7.

[38] Kleinert AMP, Eterovic A, Santos-Filho PS. Por que os levantamentos de abelhas falham quando se trata de entender suas comunidades? Polinizadores no Brasil (eds. Imperatriz-Fonseca VL, Canhos DAL, Alves DA, Saraiva AM), pp. 175–180. EDUSP, São Paulo 2012.

[39] Schneider SS, DeGrandi-Hoffman G, Smith DR. The African Honey Bee, factors contributing to a successful biological invasion. Annu Rev Entomol 2004; 49: 351–76. 14651468 10.1146/annurev.ento.49.061802.123359

[40] Giannini TC, Boff S, Cordeiro GD, Cartolano EA, Veiga AK, Imperatriz-Fonseca VL et al Crop pollinators in Brazil: a review of reported interactions. Apidologie 2015; 46: 209–223.

[41] Villanueva-Gutierrez R, Roubik DW. Why are African honey bees and not European bees invasive? Pollen diet diversity in community experiments. Apidologie 2004; 35: 481–491.

[42] Roubik DW. Ecological impact on native bees by the invasive Africanized honey bee. Acta Biológica Colombiana 2009; 14: 115–124.

[43] Carneiro LT, Martins CF. Africanized honey bees pollinate and preempt the pollen of *Spondia smombin* (Anacardiaceae) flowers. Apidologie 2012; 43: 474–486.

[44] Jordano P, Bascompte J, Olesen JM. Invariant properties in coevolutionary networks of plant-animal interactions. Ecol Lett 2003; 6: 69–81.

[45] Vasquez DP, Morris WF, Jordano P. Interaction frequency as a surrogate for the total effect of animal mutualists on plants. Ecol Lett 2005; 8: 1088–1094.

[46] Vidal MM, Hasui E, Pizo MA, Tamashiro JY, Silva WR, Guimarães JR PR. Frugivores at higher risk of extinction are the key elements of a mutualistic network. Ecology 2014; 95: 3440–3447.

[47] Sakagami S, Laroca S, Moure J. Wild bee biocenotics in Sao Jose dos Pinhais (PR), South Brazil, preliminary report. Journal of the Faculty of Sciences Hokkaido University 1967; 16: 253–291.

[48] Bascompte J, Jordano P. Plant-animal mutualistic networks: the architecture of biodiversity. Annu Rev Ecol Evol Syst 2007; 38: 567–93.

[49] Vazquez DP, Chacoff NP, Cagnolo L. Evaluating multiple determinants of the structure of plant-animal mutualistic networks. Ecology 2009; 90: 2039–2046. 19739366 10.1890/08-1837.1

[50] Legendre P, Legendre L. Ecological resemblance In: Legendre and Legendre L. Numerical Ecology: developments in environmental modelling. Amsterdam, Elsevier, p. 265–335. 2012.

[51] Bascompte J, Jordano P, Olesen JM. Asymmetric coevolutionary networks facilitate biodiversity maintenance. Science 2006; 312: 431–433. 16627742 10.1126/science.1123412

[52] Martin-Gonzales AM, Dalsgaard B, Olesen JM. Centrality measures and the importance of generalist species in pollination networks. Ecological Complexity 2010; 7: 36–41.

[53] Jordano P, Vázquez D, Bascompte J. Redes complejas de interacciones mutualistas planta-animal In: Mendel R, Aizen MA, Zamora R (eds). Ecología y evolución de interacciones planta-animal. Santiago de Chile, Universitaria p. 17–41. 2009.

[54] Almeida-Neto M, Guimarães PR Jr, Guimarães PR, Loyola RD, Ulrich W. A consistent metric for nestedness analysis in ecological systems, reconciling concept and measurement. Oikos 2008; 117: 1227–1239.

[55] Bascompte J, Jordano P, Melián CJ, Olesen JM. The nested assembly of plant–animal mutualistic networks. PNAS 2003; 100: 9383–9387. 12881488 10.1073/pnas.1633576100 PMC170927

[56] Bastolla U, Fortuna MA, Pascual-Garcia A, Ferrera A, Luque B, Bascompte J. The architecture of mutualistic networks minimizes competition and increases biodiversity. Nature 2009; 458: 1018–1020. 10.1038/nature07950 19396144

[57] Allesina S, Tang S. Stability criteria for complex ecosystems. Nature 2012; 483: 205–208. 10.1038/nature10832 22343894

[58] Burgos E, Ceva H, Perazzo RPJ, Devoto M, Medanc D, Zimmermann M et al Why nestedness in mutualistic networks? J Theor Biol 2007; 249: 307–313. 17897679 10.1016/j.jtbi.2007.07.030

[59] Fortuna MA, Bascompte J. Habitat loss and the structure of plant-animal mutualistic networks. Ecol Lett 2006; 9: 278–283. 10.1111/j.1461-0248.2005.00868.x 16958893

[60] Dormann CF, Fründ J, Blüthgen N, Gruber B. Indices, graphs and null models, analyzing bipartite ecological networks. Open Ecol J 2009; 2: 7–24.

[61] Krebs CJ. Ecological Methodology. Harper Collins, New York 1989.

[62] Hijmans R, Cameron S, Parra J, Jones P, Jarvis A. Very high resolution interpolated climate surfaces for global land areas. Int J Climatol 2005; 25: 1965–1978.

[63] R Core Team R: A Language and Environment for Statistical Computing. R Foundation for Statistical Computing http://www.R-project.org. 2013.

[64] Butts CT. Social network analysis with sna. J Stat Soft 2008; 24: 1–51.

[65] Fox J, Nie Z, Byrnes J. sem, structural equation models. 2012. Retrieved from http://cran.r-project.org/package=sem.

[66] Grace JB, Anderson TM, Olff H, Scheiner SM. On the specification of structural equation models for ecological systems. Ecol Monographs 2010; 80: 67–87.

[67] Thébault E, Fontaine C. Stability of Ecological Communities and the Architecture of Mutualistic and Trophic Networks. Science 2010; 329: 853–856. 10.1126/science.1188321 20705861

[68] Fox J. Structural Equation Modeling with the sem Package in R. Struct Equ Modeling 2006; 13: 465–486.

[69] Canard E, Mouquet N, Marescot L, Gaston K J, Gravel D, Mouillot D. Emergence of structural patterns in neutral trophic networks. PLoS ONE 2012; 7:e38295 10.1371/journal.pone.0038295 22899987 PMC3416803

[70] Canard E, Mouillot D, Mouquet N, Gravel D. Empirical evaluation of neutral interactions in host-parasite networks. Amer Nat 2014; 183: 468–479. 24642492 10.1086/675363

[71] Krishna A, Guimaraes PR. Jordano P, Bascompte J. A neutral-niche theory of nestedness in mutualistic networks. Oikos 2008; 117: 1609–1618.

[72] Cortopassi-Laurino M, Ramalho M. Pollen harvest by Africanized *Apis mellifera* and *Trigona spinipes* in São Paulo, botanical and ecological views. Apidologie 1988; 19: 1–24.

[73] Traveset A, Heleno R, Chamorro S, Vargas P, McMullen CK, Castro-Urgal R et al Invaders of pollination networks in the Galápagos Islands: emergence of novel communities. Proc R Soc Lond B 2013; 280: 30–40. 10.1098/rspb.2012.3040 PMC3619457 23486435

[74] Nieh JC, Kruizinga K, Barreto LS, Contrera FAL, Imperatriz-Fonseca VL. Effect of group size on the aggression strategy of an extirpating stingless bee, *Trigona spinipes* . Insectes Soc 2005; 52: 147–154.

[75] Jaffé R, Dietemann V, Allsopp MH, Costa C, Crewe RM, Dall’olio R et al Estimating the density of honeybee colonies across their natural range to fill the gap in pollinator decline censuses. Conserv Biol 2009; 24: 583–593. 10.1111/j.1523-1739.2009.01331.x 19775273

[76] Almeida GF. Fatores que interferem no comportamento enxameatório de abelhas africanizadas. *PhD Thesis*. Universidade de São Paulo. 2008.

[77] Girão LC, Lopes AV, Tabarelli M, Bruna EM. Changes in tree reproductive traits reduce functional diversity in a fragmented Atlantic Forest landscape. PLoSOne 2007; 2: e908. 10.1371/journal.pone.0000908 PMC1975471 17878943

