# Supplementary material for: Native and Non-Native Supergeneralist Bee Species Have Different Effects on Plant-Bee Networks
Source: PLoS One. 2015 Sep 10;10(9):e0137198. doi: 10.1371/journal.pone.0137198 (PMC4565550; doi:10.1371/journal.pone.0137198)

**S3**

**Figure 1.** Variation of a) Nestedness and degree of *Apis mellifera* (Am)*;* b) Plant niche overlap and degree of *Apis mellifera;* b) Nestedness and strength of *Apis mellifera;* d) Plant niche overlap and strength of *Apis mellifera;* e) Plant niche overlap and strength of *Trigona spinipes* (Ts); f) Bee niche overlap and strength of *Trigona spinipes*; g) Bee niche overlap and degree of *Trigona spinipes*.


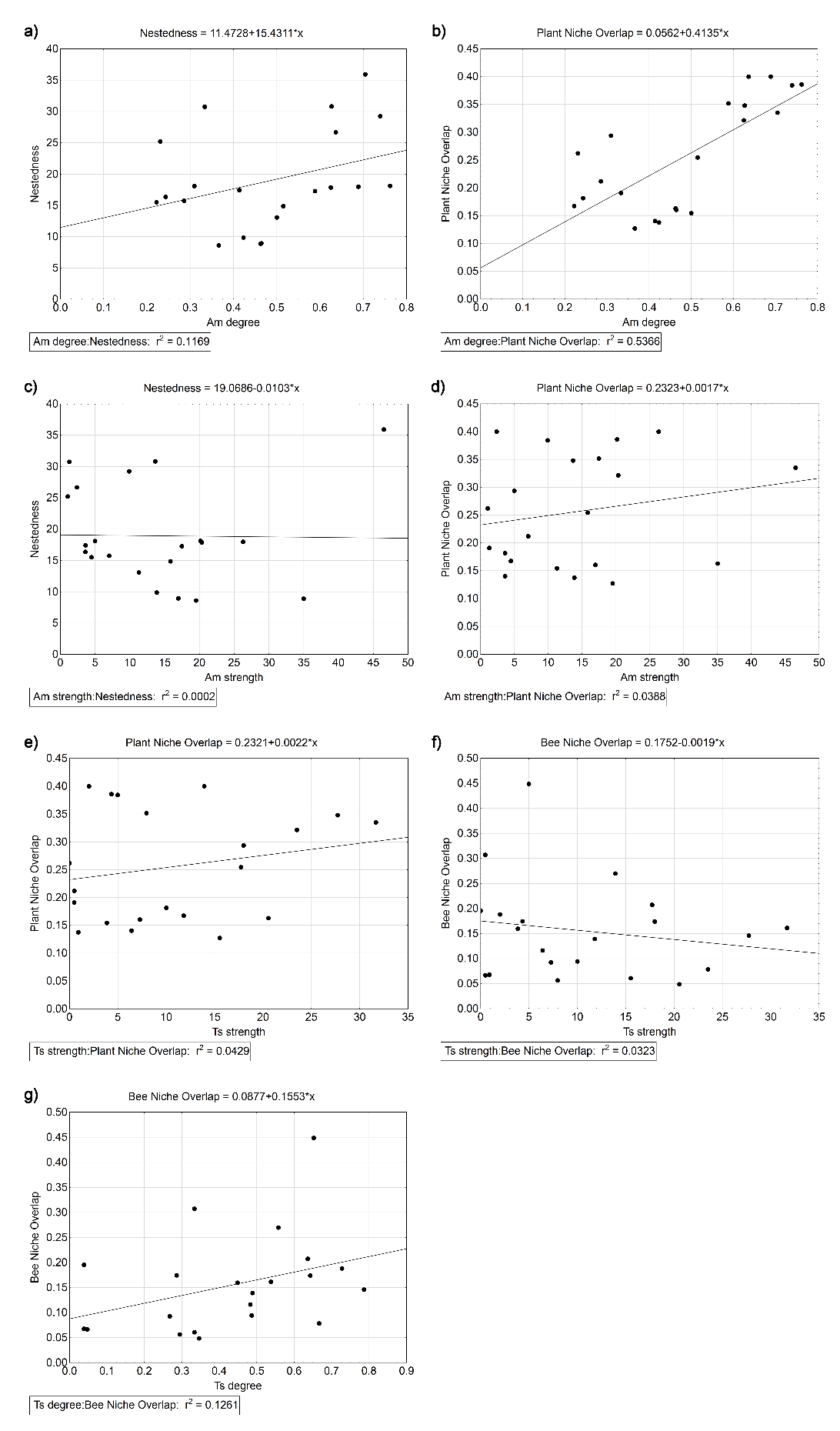

Supplement: S1 Fig — (DOCX) [file pone.0137198.s001.docx]
