# Supplementary material for: Native and Non-Native Supergeneralist Bee Species Have Different Effects on Plant-Bee Networks
Source: PLoS One. 2015 Sep 10;10(9):e0137198. doi: 10.1371/journal.pone.0137198 (PMC4565550; doi:10.1371/journal.pone.0137198)
Supplement: S1 Table — (DOCX) [file pone.0137198.s002.docx]

**S1**

**Table 1.** Data sources

| id | number of bee species | number of plant species | Reference | Habitat |
| --- | --- | --- | --- | --- |
| 1. | 23 | 37 | Aguiar 1995; Aguiar and Martins 1997 | tropical dry forest |
| 2. | 39 | 42 | Aguiar 2003; Aguiar and Zanella 2005 | tropical dry forest |
| 3. | 59 | 56 | Andena et al 2005 | tropical savanna |
| 4. | 21 | 6 | Carvalho 1993 | agroecosystem |
| 5. | 22 | 11 | Carvalho 1993; Carvalho et al 1995 | agroecosystem |
| 6. | 47 | 61 | Carvalho 1999 | tropical dry forest |
| 7. | 10 | 34 | D'Avila 2006 | tropical savanna |
| 8. | 47 | 52 | Faria 1994 | tropical savanna |
| 9. | 23 | 42 | Faria-Mucci et al 2003 | tropical savanna |
| 10. | 14 | 132 | Knoll 1990; Knoll et al 1993 | urban area |
| 11. | 73 | 66 | Lima 2004 | tropical moist forest |
| 12. | 91 | 58 | Mateus 1998 | tropical savanna |
| 13. | 124 | 123 | Pedro 1992 | tropical savanna |
| 14. | 30 | 26 | Rêgo 1998 | tropical savanna |
| 15. | 27 | 23 | Silva 1998; Silva and Martins 1999 | mangrove |
| 16. | 44 | 29 | Silveira 2006 (Costa do Sol) | tropical moist forest |
| 17. | 36 | 45 | Silveira 2006 (Agua Fria) | urban area |
| 18. | 35 | 42 | Silveira 2006 (Bairro dos Estados) | urban area |
| 19. | 43 | 72 | Sofia 1996 (Londrina) | urban area |
| 20. | 26 | 75 | Sofia 1996 (Ribeirao Preto) | urban area |
| 21. | 146 | 214 | Wilms 1995; Wilms et al 1996 | tropical moist forest |

**References**

Aguiar CML. Abundância, diversidade e fenologia de abelhas (Hymenoptera - Apoidea) da caatinga (São João do Cariri, PB) e suas interações com a flora apícola. Msc Thesis, Universidade Federal da Paraíba, João Pessoa. 1995.

Aguiar CML. Utilização de recursos florais por abelhas (Hymenoptera: Apoidea) em uma área de Caatinga (Itatim, Bahia, Brasil). Revista Brasileira de Zoologia 2003; 20: 457-467.

Aguiar CML, Martins CF. Abundância relativa, diversidade e fenologia de abelhas (Hymenoptera, Apoidea) na Caatinga, São João do Cariri, Paraíba, Brasil. Iheringia 1997; 83: 151-163.

Aguiar CML, Zanella FCV. Estrutura da comunidade de abelhas (Hymenoptera: Apoidea: Apiformis) de uma área na margem do domínio da Caatinga (Itatim, BA). Neotropical Entomology 2005; 34: 15-24.

Andena SR, Bego LR, Mechi MR. As comunidades de abelhas (Hymenoptera, Apoidea) de uma área de cerrado (Corumbataí, SP) e suas visitas às flores. Revista Brasileira Zoociências 2005; 7: 55-91.

Carvalho CAL. Abelhas (Hymenoptera, Apoidea) no município Cruz das Almas - Bahia: levantamento, identificação e material coletado em plantas de importância econômica. Msc Thesis. Universidade Federal da Bahia. Salvador. 1993.

Carvalho CAL. Diversidade de abelhas (Hymenoptera, Apoidea) e plantas visitadas no município de Castro Alves – BA. PhD Thesis. Universidade de São Paulo, Piracicaba. 1999.

Carvalho, C. A. L., Marques, O. M., Sampaio, H. S. V. Abelhas (Hymenoptera, Apoidea) em Cruz das Almas - Bahia: 1. Espécies coletadas em fruteiras. Insecta1995; 4: 11-17.

D'Avila M. Insetos visitantes florais em áreas de cerradão e cerrado sensu stricto no Estado de São Paulo. PhD Thesis. Universidade de São Paulo, Piracicaba. 2006.

Faria GM. A flora e a fauna apícola de um ecossistema de campo rupestre, Serra do Cipó – MG, Brasil: Composição, fenologia e suas interações. PhD Thesis. Universidade de São Paulo. 1994.

Faria-Mucci GM, Melo MA, Campos LAO. A fauna de abelhas (Hymenoptera, Apoidea) e plantas utilizadas como fonte de recursos florais, em um ecossistema de campos rupestres em Lavras Novas, Minas Gerais, Brasil. Apoidea Neotropica (Melo, G. R., Alves-dos-Santos, I.). UNESC, Criciúma. 2003.

Knoll FRN. Abundância relativa, sazonalidade e preferências florais de Apidae em uma área urbana (23º33’D; 46º43’W). PhD Thesis. Universidade de São Paulo, São Paulo. 1990.

Knoll FRN, Bego LR, Imperatriz-Fonseca VL. Abelhas em áreas urbanas. Um estudo no Campus da Universidade de São Paulo. Flores e abelhas em São Paulo, (Pirani, J.R. and Cortopassi-Laurino, M. eds), EDUSP, São Paulo. 1993.

Lima MFC. Comunidade de abelhas, nidificação de abelhas solitárias em cavidades preexistentes (Hymenoptera: Apoidea) e interação abelha-planta na Reserva Biológica Guaribas, Mamanguape, Paraíba, Brasil. PhD Thesis. Universidade Federal da Paraíba, João Pessoa. 2004.

Mateus S. Abundância relativa, fenologia e visita às flores pelos Apoidea do cerrado da Estação Ecológica de Jataí - Luiz Antônio – SP. Msc Thesis. Universidade de São Paulo, Ribeirão Preto. 1998.

Pedro SRM. Sobre as abelhas (Hymenoptera, Apoidea) em um ecossistema de cerrado (Cajuru, NE do Estado de São Paulo): composição, fenologia e visita às flores. Msc Thesis. Universidade de São Paulo, Ribeirão Preto. 1992.

Rêgo MMC. Abelhas silvestres (Hymenoptera, Apoidea) em um ecossistema de cerrado s.l. (Chapadinha – MA, Brasil): uma abordagem biocenótica. PhD Thesis. Universidade de São Paulo, Ribeirão Preto. 1998.

Silva MCM. Estrutura da comunidade de abelhas (Hymenoptera, Apoidea) de uma área de restinga (Praia de Intermares, Cabedelo, Paraíba, Nordeste do Brasil). Msc Thesis. Universidade Federal da Paraíba, João Pessoa. 1998.

Silva MCM, Martins CF. Flora apícola e relações tróficas de abelhas (Hymenoptera, Apoidea) em uma área de restinga (Praia de Intermares, Cabedelo - PB, Brasil). Principia 1999; 7: 40-51.

Silveira MS. Fauna de abelhas (Hymenoptera, Apoidea, Apiformes) e recursos florais utilizados em áreas urbanas e no entorno da cidade de João Pessoa, PB. Msc Thesis. Universidade Federal da Paraíba, João Pessoa. 2006.

Sofia SH. As abelhas e suas visitas às flores em duas áreas urbanas. PhD Thesis. Universidade Estadual Paulista, Rio Claro. 1996.

Wilms W. Die Bienenfauna im Küstenregenwald Braziliens und ihre Beziehungen zu Blütenpflanzen: Fallstudie Boracéia, São Paulo. PhD Thesis. Universität Tübingen, Tübingen. 1995.

Wilms W, Imperatriz-Fonseca VL, Engels W. Resource partitioning between highly eusocial bees and possible impact of the introduced Africanized honey bee on native stingless bees in the Brazilian Atlantic Rainforest. Studies on Neotropical Fauna and Environment 1996: 31: 137-151.
