# Supplementary material for: Native and Non-Native Supergeneralist Bee Species Have Different Effects on Plant-Bee Networks
Source: PLoS One. 2015 Sep 10;10(9):e0137198. doi: 10.1371/journal.pone.0137198 (PMC4565550; doi:10.1371/journal.pone.0137198)
Supplement: S2 Table — (DOCX) [file pone.0137198.s003.docx]

**S2**

**Table 1.** Pearson's correlation coefficients between total number of individual sampled, total richness, and the network indexes employed in our study. Significant correlations are shown in bold. (Am = *Apis mellifera;* Ts = *Trigona spinipes;* NO= niche overlap).

|  | plant richness | connectance | NODF | plant NO | bee NO | Am strength | Ts strength | Am degree | Ts degree | Am abundance | Ts abundance | other bee abundance | altitude | precipitation | temperature |
| --- | --- | --- | --- | --- | --- | --- | --- | --- | --- | --- | --- | --- | --- | --- | --- |
| bee richness | **0.72** | **-0.66** | **-0.65** | **-0.50** | -0.30 | 0.34 | 0.18 | -0.10 | -0.10 | 0.30 | -0.12 | **0.53** | 0.30 | 0.33 | -0.34 |
| p value | *0.0003* | *0.0013* | *0.0014* | *0.0205* | 0.1802 | 0.1329 | 0.4366 | 0.6800 | 0.6684 | 0.1868 | 0.5954 | *0.0129* | 0.1890 | 0.1442 | 0.1278 |
| plant richness |  | -0.31 | -0.27 | -0.21 | -0.41 | **0.79** | **0.65** | 0.12 | 0.02 | **0.59** | 0.31 | **0.78** | **0.49** | 0.35 | **-0.62** |
| p value |  | 0.1702 | 0.2317 | 0.3642 | 0.0678 | *0.00002* | *0.0013* | 0.5952 | 0.9483 | *0.0048* | 0.1712 | *0.00003* | *0.0227* | 0.1228 | *0.0025* |
| connectance |  |  | **0.97** | **0.45** | **0.52** | -0.09 | 0.06 | 0.22 | 0.28 | -0.02 | 0.32 | 0.06 | -0.31 | -0.09 | 0.23 |
| p value |  |  | *1.0E-12* | *0.0386* | *0.0150* | 0.6930 | 0.7954 | 0.3328 | 0.2117 | 0.9427 | 0.1515 | 0.7893 | 0.1743 | 0.7031 | 0.3240 |
| NODF |  |  |  | **0.60** | **0.60** | -0.02 | 0.17 | 0.34 | 0.39 | 0.13 | 0.43 | 0.14 | -0.38 | -0.11 | 0.29 |
| p value |  |  |  | *0.0043* | *0.0042* | 0.9480 | 0.4610 | 0.1293 | 0.0812 | 0.5733 | 0.0507 | 0.5540 | 0.0925 | 0.6426 | 0.1948 |
| plant NO |  |  |  |  | **0.47** | 0.20 | 0.21 | **0.73** | **0.47** | 0.37 | 0.33 | 0.004 | -0.33 | -0.31 | 0.20 |
| p value |  |  |  |  | *0.0331* | 0.3920 | 0.3678 | *0.0002* | *0.0307* | 0.0976 | 0.1500 | 0.9855 | 0.1477 | 0.1777 | 0.3807 |
| bee NO |  |  |  |  |  | -0.19 | -0.18 | 0.33 | 0.36 | 0.16 | -0.07 | -0.11 | **-0.65** | -0.08 | **0.58** |
| p value |  |  |  |  |  | 0.4051 | 0.4359 | 0.1455 | 0.1142 | 0.4808 | 0.7657 | 0.6404 | *0.0014* | 0.7220 | *0.0058* |
| Am strength |  |  |  |  |  |  | **0.66** | **0.55** | 0.07 | **0.82** | 0.35 | **0.55** | **0.44** | 0.02 | **-0.61** |
| p value |  |  |  |  |  |  | *0.0012* | *0.0094* | 0.7596 | *5.5E-06* | 0.1199 | *0.0096* | *0.0486* | 0.9382 | *0.0031* |
| Ts strength |  |  |  |  |  |  |  | 0.30 | **0.57** | **0.62** | **0.81** | **0.69** | 0.09 | 0.16 | -0.23 |
| p value |  |  |  |  |  |  |  | 0.1894 | *0.0066* | *0.0030* | *9.0E-06* | *0.0005* | 0.7082 | 0.4792 | 0.3180 |
| Am degree |  |  |  |  |  |  |  |  | **0.45** | **0.61** | 0.33 | 0.22 | -0.03 | -0.23 | -0.16 |
| p value |  |  |  |  |  |  |  |  | *0.0424* | *0.0032* | 0.1408 | 0.3457 | 0.9136 | 0.3207 | 0.4858 |
| Ts degree |  |  |  |  |  |  |  |  |  | 0.34 | **0.57** | 0.32 | **-0.47** | -0.01 | 0.30 |
| p value |  |  |  |  |  |  |  |  |  | 0.1264 | *0.0068* | 0.1629 | *0.0301* | 0.9749 | 0.1828 |
| Am abundance |  |  |  |  |  |  |  |  |  |  | 0.39 | **0.59** | 0.13 | -0.12 | -0.30 |
| p value |  |  |  |  |  |  |  |  |  |  | 0.0825 | *0.0050* | 0.5637 | 0.5922 | 0.1810 |
| Ts abundance |  |  |  |  |  |  |  |  |  |  |  | **0.62** | 0.08 | 0.06 | -0.13 |
| p value |  |  |  |  |  |  |  |  |  |  |  | *0.0026* | 0.7255 | 0.8092 | 0.5606 |
| other bee abundance |  |  |  |  |  |  |  |  |  |  |  |  | 0.23 | 0.29 | -0.32 |
| p value |  |  |  |  |  |  |  |  |  |  |  |  | 0.3251 | 0.2076 | 0.1602 |
| altitude |  |  |  |  |  |  |  |  |  |  |  |  |  | 0.02 | **-0.93** |
| p value |  |  |  |  |  |  |  |  |  |  |  |  |  | 0.9232 | *6.08E-10* |
| precipitation |  |  |  |  |  |  |  |  |  |  |  |  |  |  | -0.04 |
| p value |  |  |  |  |  |  |  |  |  |  |  |  |  |  | 0.8557 |
| temperature |  |  |  |  |  |  |  |  |  |  |  |  |  |  |  |
| p value |  |  |  |  |  |  |  |  |  |  |  |  |  |  |  |
